# Significance of SUV Max for Predicting Occult Lymph Node Metastasis and Prognosis in Early-Stage Tongue Squamous Cell Carcinoma

**DOI:** 10.1155/2020/6241637

**Published:** 2020-03-30

**Authors:** Chunmiao Xu, Hailiang Li, Dongjie Seng, Fei Liu

**Affiliations:** ^1^Department of Radiology, Affiliated Cancer Hospital of Zhengzhou University, Henan Cancer Hospital, Zhengzhou, China; ^2^Department of Otorhinolaryngology, Children's Hospital to Zhengzhou University, Zhengzhou University, Zhengzhou, China; ^3^Department of Oral Medicine, The First Affiliated Hospital of Zhengzhou University, Zhengzhou, China

## Abstract

**Objective:**

Our goal was to clarify the significance of SUV max for predicting occult lymph node metastasis and prognosis in early-stage tongue squamous cell carcinoma (SCC).

**Methods:**

cT1-2N0 tongue SCC patients who underwent a preoperative PET-CT examination were prospectively enrolled. The association between SUV max and occult lymph node metastasis was analyzed. The main study endpoint was locoregional control (LRC). The Cox model was used to determine the independent factors.

**Results:**

A total of 120 patients were included for analysis, and the median SUV max was 9.7. In 60 patients with an SUV max ≤9.7, 5 patients had occult metastasis; in 60 patients with an SUV max >9.7, 13 patients had occult metastasis, and the difference was significant (*p*=0.041). In patients with an SUV max ≤9.7, the 5-year LRC rate was 93%; in patients with an SUV max >9.7, the 5-year LRC rate was 81%, and the difference was significant (*p*=0.045).

**Conclusion:**

An SUV max >9.7 was a marker for occult lymph node metastasis and could decrease LRC in patients with cT1-2N0 tongue SCC.

## 1. Introduction

Neck lymph node status is the most important prognostic factor in oral squamous cell carcinoma (SCC), and survival usually decreases by half once there is lymph node metastasis. Owing to the various lymph node metastasis phenomena, there is great controversy regarding the neck management of cT1-2N0 disease patients. Researchers who support routine neck dissection describe that it can select patients who need adjuvant radiotherapy and then improve survival [1, 2], but others argue that most patients with cT1-2N0 disease do not have pathologic lymph node metastasis; they are overtreated and exposed to possible neck dissection-related complications [3, 4]. Therefore, it is important for us to identify reliable predictors of neck lymph node metastasis, and current evidence supports the predictive value of depth of invasion (DOI), perineural invasion (PNI), and lymphovascular invasion (LVI). However, these data are not always able to be obtained preoperatively.

PET-CT has been widely used to assess metastatic disease and primary sites by analyzing the value of maximum standardized uptake (SUV max). In general, FDG is more likely to be taken up by cancers with high proliferative ability, and SUV max is a reliable indicator of FDG accumulation. Hasegawa et al. [5] noted that an SUV max greater than 8.0 was significantly associated with tumor stage, PNI, LVI, and Ki-67 expression in oral SCC. However, tongue SCC shows different biologic behavior compared to other subsites of SCC; moreover, whether SUV max can be used as a marker for occult lymph node metastasis in early-stage tongue SCC remains unknown. Therefore, in the current study, we aimed to clarify the significance of SUV max in predicting occult lymph node metastasis and prognosis in early-stage tongue SCC.

## 2. Patients and Methods

The Zhengzhou University Institutional Research Committee approved our study, and all patients signed informed consent agreements for medical research before the initial treatment. All methods were performed in accordance with the relevant guidelines and regulations.

From January 2008 to December 2015, patients with primary early-stage (cT1-2N0) tongue SCC were prospectively enrolled. The only inclusion criterion was that the patient agreed to have a PET-CT examination preoperatively. Clinical pathologic and follow-up data, including age, sex, smoking status, drinking status, pathologic TNM stage based on the AJCC 8^th^ edition, perineural invasion (PNI), lymphovascular invasion (LVI), extracapsular extension (ECS), depth of invasion (DOI), SUV max of the primary tumor, and SUV max of the lymph node, were recorded for the enrolled patients.

PET-CT (GE Healthcare, Milwaukee, America) was manipulated by several technicians. Before the PET/CT scan, patients fasted for at least 6 hours and were instructed to avoid strenuous exercise. Imaging was put off if glucose levels were >200 mg/dL. Each patient received 10–20 mCi of [^18^F] FDG dosed according to body weight. Axial PET and diagnostic CT images were obtained from the calvarial vertex through the upper thighs after urinary voiding. Emission images were obtained 60 minutes after radiopharmaceutical injection. No contrast medium was used during the CT scan. The images were reconstructed in the thickness of 2.5 mm slice. SUV max was measured for both the primary tumor and regional lymph nodes. For every suspicious lesion, the isocontour region of interest centered on the maximum value pixel was drawn automatically with workstation tools generating the SUV max of the region. An SUV max cutoff of 2.5 MBq/g was used for FDG-avid lymph nodes and primary tumors on PET-CT.

Smokers/drinkers were defined as patients who smoked/drank at diagnosis or who had stopped smoking/drinking for less than 1 year [6, 7]. All pathologic sections were reviewed by at least two pathologists. PNI was considered to be present if tumor cells were identified within the perineural space and/or nerve bundle; LVI was positive if a tumor was noted within the lymphovascular channels [8]. The pathologic DOI was measured from the level of the adjacent normal mucosa to the deepest point of tumor infiltration, regardless of the presence or absence of ulceration [9]. cT1-2 was defined as a maximum diameter of tumors less than 2 cm or a diameter ranging from 2 cm to 4 cm. Patients were considered to have cN0 disease if they had no evidence of nodal metastasis on clinical examination, ultrasound, or radiographic imaging (not including PET-CT) [10]. The cutoff value of SUV max was set according to the median value [11].

In our cancer center, systemic examinations, including ultrasound, CT, and MRI, were routinely performed, PET-CT was selectively suggested, and complete resection of the primary tumor with a margin of at least 1 cm, including the adipose tissue in the floor of the mouth as well as sublingual gland and neck dissection (level 1–3), was routinely performed for every patient with any stage of tongue SCC. Adjuvant treatment was suggested if there was neck lymph node metastasis, PNI, LVI, or a positive margin. All patients were regularly followed up every 3 months within the first two years after the operation and every 6 months within the third to fifth years after the operation. If there was any doubt regarding disease recurrence, active interference was performed.

The primary study interests were occult neck lymph node metastasis and locoregional control (LRC). LRC referred to the percentage of people who had locoregional recurrence in a defined period of time. The Chi-square test was used to analyze the association between clinical pathologic variables and occult lymph node metastasis. The factors that were significant in the Chi-square test were then analyzed with multilogistic regression to determine independent predictors. The survival time was calculated from the date of surgery to the date of the first event of local recurrence, regional recurrence, or locoregional recurrence or to the date of the latest follow-up. The Kaplan-Meier method was used to analyze the LRC rate, and then the factors that were significant according to the Kaplan-Meier method were assessed in the Cox model to determine the independent factors. All statistical analyses were performed using SPSS 20.0, and *p* < 0.05 was considered to be significant.

## 3. Results

A total of 120 patients (91 males and 29 females) were ultimately enrolled, and the median age was 60 years, with a range from 31 to 75 years. cT1 tumors were present in 49 (40.8%) patients, and cT2 tumors were present in 71 (59.2%) patients. A total of 86 (71.7%) and 62 (51.7%) patients were smokers and drinkers, respectively. The median SUV max was 9.7, with a range from 2.8 to 24.9.

The median DOI was 4.6 mm, with a range from 1.8 mm to 9.2 mm; pT1 tumors were present in 43 (35.8%) patients, and pT2 tumors were present in 77 (64.2%) patients. Cancer cells showed good differentiation in 42 (35.0%) patients, moderate differentiation in 63 (52.5%) patients, and poor differentiation in 15 (12.5%) patients. PNI was reported in 16 (13.3%) patients, and LVI was reported in 14 (11.7%) patients. Negative margins were obtained in all patients. Occult lymph node metastasis was reported in 18 (15.0%) patients, ECS was reported in 2 (2/18, 11.1%) patients, and the total positive lymph node number was 22.

The associations between clinical variables and occult lymph node metastasis are shown in Tables 1 and 2. In univariate analysis, the factors of SUV max, pathologic tumor stage, and tumor differentiation were significantly associated with the occurrence of occult metastasis (all *p* < 0.05), and no apparent relationships between other clinical pathologic variables and the occurrence of occult metastasis were noted (all *p* > 0.05). Further multivariate analyses confirmed that high SUV max, high pathologic tumor stage, and poor tumor differentiation were independent factors for an increased risk of occult metastasis.

A total of 22 patients were reported to have lymph node metastasis according to preoperative PET-CT, of whom 14 patients had pathologic metastasis; 98 patients were reported to have no lymph node metastasis according to preoperative PET-CT, of whom 4 patients had pathologic metastasis. The sensitivity and specificity of PET-CT for predicting occult metastasis were 77.8% and 92.2%, respectively.

**Figure 1:**
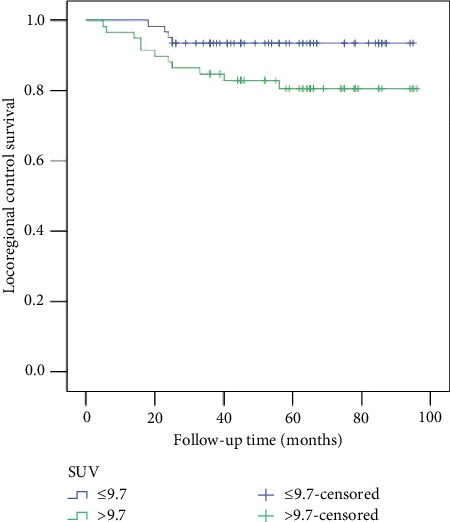
Locoregional control in patients with different SUV max values (*p*=0.045).

During our follow-up with a median time of 55 months, 15 patients developed recurrence: locally in 5 cases, regionally in 5 cases, and locoregionally in 5 cases. Eight patients died from the cancer. The 5-year LRC rate was 87%. In patients with an SUV max ≤9.7, the 5-LRC rate was 93%; in patients with an SUV max >9.7, the 5-year LRC rate was 81%, and the difference was significant (Figure 1, *p*=0.045). The Cox model further confirmed that LRC was an independent factor affecting occult metastasis (Table 3).

## 4. Discussion

The most significant finding in the current study was that an SUV max >9.7 could not only increase the possibility of neck lymph node metastasis but also predict worse LRC. This finding may improve decision-making for neck management when facing cT1-2N0 tongue SCC and may be suggestive of adjuvant treatment when there is a high SUV max.

PET-CT is currently the most advanced medical equipment used for detecting metastatic sites and staging oncology diseases. A number of current researchers have reported a higher sensitivity and specificity of PET-CT than of MRI and CT for determining positive lymph nodes [12, 13]. Piao et al. [14] previously demonstrated that the sensitivity and specificity of PET-CT for detecting the neck metastatic region were 84% and 87%, respectively, by using an SUV max cutoff value of 2.5 in oral SCC. McGuirt et al. [15] described a sensitivity and specificity of 75% and 63.8%, respectively, using the same threshold value. Similar results were also reported by Liao et al. [16], whose study consisted of 473 oral SCC patients with an SUV max threshold value of 3.1. All these findings support the high reliability of PET-CT for identifying metastatic lymph nodes. However, these studies failed to answer the question of whether SUV max could be used as a marker for occult lymph node metastasis.

A few authors have evaluated the significance of SUV max in oral SCC. Yamada et al. [17] noted that the SUV max in oral SCC was only significantly associated with the tumor stage but not with other important pathologic variables, including the neck lymph node stage and cancer cell differentiation. Upon further analysis, the relationship between FDG uptake and the expression levels of GLUT-1, HK II, and HIF-1*α* was reported in early-stage oral SCC but not in advanced-stage disease. However, in the study by Hasegawa et al. [5], the authors found that cT stage, cN stage, infiltrative pattern, PNI, and Ki-67 expression were significantly correlated with the SUV max in univariable analysis. However, the authors enrolled patients with oral SCC without focusing on tongue SCC patients, and tongue SCC shows different biologic behaviors than SCC at other sites. Moreover, a multivariate analysis was not performed to alleviate the effect of confounding factors. Zhang et al. [18] might be the first to assess the role of PET-CT in evaluating cN0 in early-stage tongue SCC; the authors described that the overall sensitivity and specificity of PET-CT in their patients were 21.4% and 98.4%, respectively, with a negative predictive value of 99.1%. Although the sensitivity improved in patients with tumors ≥2 cm in size and ≥4 mm in depth, the specificity remained unchanged. However, the authors did not analyze the relationship between SUV max and neck lymph node metastasis. We were the first to find that an SUV max >9.7 carries an additional 2-fold risk for occult metastasis. This finding might be attributed to the fact that a high SUV max was associated with strong invasiveness and growth potential.

The prognostic factors for tongue SCC have been extensively analyzed. The widely accepted risk factors include high tumor stage, neck lymph node metastasis, PNI, and LVI. In the current study, we found that an SUV max >9.7 was associated with worse LRC. Yokobori et al. [19] described that SUV max was an independent prognostic factor for progression-free survival in patients undergoing chemoradiotherapy for tongue SCC with a cutoff value of 5.0. In a previous study, Liao et al. [20] reported that their optimal cutoff value for SUV max was 5.7, and an SUV max >5.7 indicated a worse 5-year neck cancer control rate, distant metastatic rate, and disease-specific survival. All these findings support that a high SUV max might cause worse disease survival, and more adjuvant treatment is needed. Other significant prognostic factors in the current study included LVI, cervical lymph node metastasis, and tumor differentiation. This finding was consistent with previous studies [21–23]. Poor tumor differentiation was indicated by a higher Ki-67 index, a higher SUV max value, and an enhanced invasion ability of cancer cells [24]. The frequency of LVI ranges from 10% to 81% in oral SCC, and LVI is significantly related to tumor primary size and pT stage [25].

The greatest strength of our study was the prospective design, which provided high reliability for comprehending our results. We hope the study will benefit future cN0 neck management in cT-2 tongue SCC. However, some limitations of the current study must be acknowledged: our sample size was small, there might be the risk of overparameterisation in multivariate model, and some underlying factors remained unknown, such as the molecular mechanism.

In summary, an SUV max >9.7 was a marker for occult lymph node metastasis and could decrease LRC in patients with cT1-2N0 tongue SCC.

## Figures and Tables

**Table 1:** Univariate analysis of the association between clinical pathologic variables and occult lymph node metastasis in cT1-2N0 tongue squamous cell carcinoma patients.

Clinical pathologic variables	Occult metastasis	Chi-test	*p*
	Positive (*n* = 18)	Negative (*n* = 102)	
Age			
≤40	3	10	
>40	15	92	0.411
Sex			
Male	14	77	
Female	4	25	1.000
Smoking			
Yes	14	72	
No	4	30	0.533
Drinking			
Yes	10	52	
No	8	50	0.720
SUV max			
≤9.7	5	55	
>9.7	13	47	0.041
Clinical tumor stage			
cT1	4	45	
cT2	14	57	0.118
Pathologic tumor stage			
pT1	2	41	
pT2	16	61	0.030
Perineural invasion			
Yes	4	12	
No	14	90	0.259
Lymphovascular invasion			
Yes	3	11	
No	15	91	0.691
Tumor differentiation			
Well	3	39	
Moderate	10	53	
Poor	5	10	0.046

**Table 2:** Multilogistic regression of the association between clinical pathologic variables and occult lymph node metastasis in cT1-2N0 tongue squamous cell carcinoma patients.

Clinical pathologic variables	Multilogistic regression		
	*p*	OR	95% CI
SUV max	0.043	2.879	1.264–5.997
Pathologic tumor stage	0.007	4.658	2.667–9.678
Tumor differentiation			
Well			
Moderate	0.016	3.699	1.876–8.338
Poor	<0.001	6.668	2.447–20.665

**Table 3:** Univariate and multivariate Cox model analyses of risk factors for the locoregional control survival in patients with cT1-2N0 tongue squamous cell carcinoma.

Variables	Univariate		Cox model	
	*p*	HR (95% CI)	*p*	HR (95% CI)
Age (<40 vs. ≥40)	0.621	1.395 (0.684–3.678)		
Gender	0.463	2.003 (0.771–6.874)		
Smoking	0.555	2.224 (0.412–8.663)		
Drinking	0.746	1.228 (0.229–9.007)		
SUV max (≤9.7 vs. >9.7)	0.045	3.445 (1.223–8.247)	0.034	2.472 (1.445–4.752)
Pathologic tumor stage (T1 vs. T2)	0.022	5.221 (1.888–12.521)	0.568	4.612 (0.741–9.667)
Pathologic neck lymph node stage (N0 vs. N+)	0.004	6.221 (1.997–15.322)	<0.001	4.668 (1.964–9.972)
Perineural invasion	0.069	2.665 (0.975–6.335)		
Extracapsular extension	0.785	3.556 (0.639–8.554)		
Lymphovascular invasion	0.042	2.334 (1.547–7.338)	0.031	2.558 (1.487–5.221)
Tumor differentiation	0.011	4.557 (2.006–13.332)		
Well				
Moderate			0.152	2.667 (0.856–6.442)
Poor			0.006	3.978 (1.997–9.331)
Radiotherapy	0.641	0.885 (0.437–3.669)		

## Data Availability

All primary data are available from the corresponding author upon reasonable request.

## References

[1] Canis M., Plüquett S., Ihler F., Matthias C., Kron M., Steiner W. (2012). Impact of elective neck dissection vs observation on regional recurrence and survival in cN0-staged patients with squamous cell carcinomas of the upper aerodigestive tract. *Archives of Otolaryngology-Head & Neck Surgery*.

[2] Borgemeester M. C., van den Brekel M. W. M., van Tinteren H. (2008). Ultrasound-guided aspiration cytology for the assessment of the clinically N0 neck: factors influencing its accuracy. *Head & Neck*.

[3] D’Cruz A. K., Vaish R., Kapre N. (2015). Elective versus therapeutic neck dissection in node-negative oral cancer. *The New England Journal of Medicine*.

[4] Liu J. Y., Chen C. F., Bai C. H. (2019). Elective neck dissection versus observation in early-stage (cT1/T2N0) oral squamous cell carcinoma. *Laryngoscope Investigative Otolaryngology*.

[5] Hasegawa O., Satomi T., Kono M., Watanabe M., Ikehata N., Chikazu D. (2019). Correlation between the malignancy and prognosis of oral squamous cell carcinoma in the maximum standardized uptake value. *Odontology*.

[6] Ouyang P.-Y., Su Z., Mao Y.-P., Liang X.-X., Liu Q., Xie F.-Y. (2013). Prognostic impact of family history in southern Chinese patients with undifferentiated nasopharyngeal carcinoma. *British Journal of Cancer*.

[7] Fang Q.-G., Shi S., Liu F.-Y., Sun C.-F. (2014). Squamous cell carcinoma of the oral cavity in ever smokers: a matched-pair analysis of survival. *Journal of Craniofacial Surgery*.

[8] Fang Q., Li P., Qi J., Luo R., Chen D., Zhang X. (2019). Value of lingual lymph node metastasis in patients with squamous cell carcinoma of the tongue. *The Laryngoscope*.

[9] Skulsky S. L., O’Sullivan B., McArdle O. (2017). Review of high-risk features of cutaneous squamous cell carcinoma and discrepancies between the American Joint Committee on Cancer and NCCN Clinical Practice Guidelines in Oncology. *Head & Neck*.

[10] Lydiatt W. M., Patel S. G., O’Sullivan B. (2017). Head and Neck cancers-major changes in the American Joint Committee on Cancer Eighth Edition Cancer staging Manual. *CA: A Cancer Journal for Clinicians*.

[11] Halfpenny W., Hain S. F., Biassoni L., Maisey M. N., Sherman J. A., McGurk M. (2002). FDG-PET. A possible prognostic factor in head and neck cancer. *British Journal of Cancer*.

[12] Gordin A., Golz A., Keidar Z., Daitzchman M., Bar-Shalom R., Israel O. (2007). The role of FDG-PET/CT imaging in head and neck malignant conditions: impact on diagnostic accuracy and patient care. *Otolaryngology-Head and Neck Surgery*.

[13] Jeong H.-S., Baek C.-H., Son Y.-I. (2007). Use of integrated ^18^F-FDG PET/CT to improve the accuracy of initial cervical nodal evaluation in patients with head and neck squamous cell carcinoma. *Head & Neck*.

[14] Piao Y., Bold B., Tayier A. (2009). Evaluation of ^18^F-FDG PET/CT for diagnosing cervical nodal metastases in patients with oral cavity or oropharynx carcinoma. *Oral Surgery, Oral Medicine, Oral Pathology, Oral Radiology, and Endodontology*.

[15] McGuirt W. F., Williams D. W., Keyes J. W. (1995). A comparative diagnostic study of head and neck nodal metastases using positron emission tomography. *The Laryngoscope*.

[16] Liao C.-T., Wang H.-M., Huang S.-F. (2011). PET and PET/CT of the neck lymph nodes improves risk prediction in patients with squamous cell carcinoma of the oral cavity. *Journal of Nuclear Medicine*.

[17] Yamada T., Uchida M., Kwang-Lee K. (2012). Correlation of metabolism/hypoxia markers and fluorodeoxyglucose uptake in oral squamous cell carcinomas. *Oral Surgery, Oral Medicine, Oral Pathology and Oral Radiology*.

[18] Zhang H., Seikaly H., Biron V. L., Jeffery C. C. (2018). Utility of PET-CT in detecting nodal metastasis in cN0 early stage oral cavity squamous cell carcinoma. *Oral Oncology*.

[19] Yokobori Y., Toyoda M., Sakakura K., Kaira K., Tsushima Y., Chikamatsu K. (2015). ^18^F-FDG uptake on PET correlates with biological potential in early oral squamous cell carcinoma. *Acta Oto-Laryngologica*.

[20] Liao C.-T., Chang J. T.-C., Wang H.-M. (2009). Preoperative [^18^F]fluorodeoxyglucose positron emission tomography standardized uptake value of neck lymph nodes predicts neck cancer control and survival rates in patients with oral cavity squamous cell carcinoma and pathologically positive lymph nodes. *International Journal of Radiation Oncology ∗ Biology ∗ Physics*.

[21] Cui M., Du W., Fang Q., Dai L., Qi J., Luo R. (2019). Prognostic value of a family history of oral tongue squamous cell carcinoma: a matched-pair study. *The Laryngoscope*.

[22] Du W., Fang Q., Wu Y., Wu J., Zhang X. (2019). Oncologic outcome of marginal mandibulectomy in squamous cell carcinoma of the lower gingiva. *BMC Cancer*.

[23] Dai L., Fang Q., Li P., Wu J., Zhang X. (2020). Secondary squamous cell carcinoma of the oral cavity after nasopharyngeal carcinoma. *Cancer Research and Treatment*.

[24] Surov A., Meyer H. J., Höhn A.-K., Winter K., Sabri O., Purz S. (2019). Associations between [^18^F]FDG-PET and complex histopathological parameters including tumor cell count and expression of KI 67, EGFR, VEGF, HIF-1*α*, and p53 in head and neck squamous cell carcinoma. *Molecular Imaging and Biology*.

[25] Chang W.-C., Chang C.-F., Li Y.-H. (2019). A histopathological evaluation and potential prognostic implications of oral squamous cell carcinoma with adverse features. *Oral Oncology*.

